# MicroRNA, MicroRNA-lncRNA and MicroRNA-Circular RNA axes, and exosomal MicroRNAs: driving exercise-induced cardioprotection in heart failure

**DOI:** 10.3389/fcell.2026.1767057

**Published:** 2026-03-25

**Authors:** Yang Li, Junmin Wang, De Ma, Jinpeng He

**Affiliations:** 1 College of P. E and Sports, Beijing Normal University, Beijing, China; 2 College of Physical Education, Zhangjiakou University, Zhangjiakou, Hebei, China; 3 School of Sports and Health Engineering, Hebei University of Engineering, Handan, China

**Keywords:** biomarkers, cardioprotection, circular RNAs, exercise, exerkines, exosomes, long non-coding RNAs, MicroRNAs

## Abstract

Regular physical activity is a powerful non-pharmacological strategy for preventing and managing cardiovascular diseases (CVDs), including heart failure, by promoting cardioprotective adaptations through molecular mechanisms that remain incompletely elucidated. This review explores the central role of non-coding RNAs (ncRNAs), particularly microRNAs (miRNAs), in exercise-induced cardioprotection, highlighting their interactions within miRNA-lncRNA and miRNA-circRNA axes, as well as the function of exosomal miRNAs as key exerkines facilitating inter-organ crosstalk. Synthesizing current literature, we examine ncRNA biogenesis, canonical functions, and exercise-responsive profiles, focusing on pivotal miRNAs such as miR-1, miR-133, miR-21, miR-126, miR-29, miR-208a, and miR-499; lncRNA-miRNA networks including MALAT1/miR-150-5p, H19/miR-139, and GAS5/miR-217; circRNA-miRNA interactions like circUtrn/miR-132/212; and exosomal miRNAs derived from skeletal muscle (e.g., miR-130a, miR-1), brown adipose tissue (e.g., miR-17-3p), endothelium (e.g., miR-126), and cardiomyocytes (e.g., miR-21-3p). These elements are evaluated in models of physiological cardiac remodeling, myocardial infarction, ischemia-reperfusion injury, diabetic cardiomyopathy, and heart failure, with consideration of influencing factors such as sex, age, and training modality. Exercise-modulated miRNAs differentiate benign “athlete’s heart” from pathological hypertrophy by governing angiogenesis, fibrosis, metabolic shifts, and arrhythmia risk, while lncRNA-miRNA and circRNA-miRNA axes regulate apoptosis, inflammation, mitochondrial dynamics, and extracellular matrix remodeling in CVD contexts. Exosomal miRNAs enable remote protection by activating survival, angiogenic, and anti-fibrotic pathways via signaling cascades like PI3K/AKT and NF-κB. Responses exhibit variability based on demographic and exercise variables, underscoring ncRNAs' promise as diagnostic biomarkers, therapeutic targets, or mimics of exercise benefits for heart failure management.

## 1 Introduction

Cardiovascular diseases (CVDs) remain the leading cause of morbidity and mortality globally, despite enormous progress in our understanding and treatment of these conditions (Bye et al., 2013; Naveed et al., 2022). Success in managing acute events, such as myocardial infarction (MI), has resulted in a growing population of survivors who face a continued risk of late sequelae, including heart failure (HF) and arrhythmia, for which effective long-term therapies remain inadequate (Liu et al., 2017; Moras et al., 2024). In the context of this persistent clinical challenge, the investigation into the mechanisms of exercise-induced cardioprotection offers a promising avenue for novel therapeutic strategies (Bernardo et al., 2018).

Regular physical activity is an effective and safe non-pharmacological intervention, capable of reducing CVD prevalence and lowering death rates in both healthy individuals and in patients with established disease (Fernandes et al., 2015; Qian et al., 2024). The beneficial effects of exercise training manifest through a variety of physiological and anatomical adaptations, collectively known as the “athlete’s heart” (Liu et al., 2017; Jakovljevic et al., 2024; La Gerche et al., 2022). These adaptations include an increase in cardiac angiogenesis, improved vascular function, and a reduction in both systolic and diastolic blood pressure (Fernandes et al., 2015; Hellsten and Nyberg, 2016). The molecular underpinnings of these protective effects, however, are still not fully understood, though they are known to involve complex signaling pathways that mediate gene expression changes in the heart (Golbidi and Laher, 2011; Clerk et al., 2007; Schirone et al., 2017).

In recent years, microRNAs have emerged as critical regulators of gene expression, adding a crucial layer of post-transcriptional control to the complex network of cellular signaling (Polakovičová et al., 2016; Aghabozorg Afjeh and Ghaderian, 2013). As small, non-coding RNA molecules, miRNAs modulate fundamental biological processes such as cell proliferation, metabolism, and apoptosis (Gao et al., 2023; Santosh et al., 2015). It is now widely accepted that the dysregulation of miRNAs is associated with the development of various diseases, including CVD, and that they are sensitive mediators of the body’s response to environmental stimuli and stress (Gao et al., 2023; Sessa et al., 2023). This review will examine the mounting evidence that miRNAs are central executors of the adaptive changes induced by exercise, exploring their pivotal role in mediating cardioprotection and their significant potential as novel diagnostic and therapeutic tools for cardiovascular health (Gao et al., 2023).

## 2 The molecular machinery: from miRNA biogenesis to inter-organ communication

### 2.1 Foundational principles of miRNA function

MiRNAs are small, evolutionarily conserved, single-stranded, non-coding RNAs typically composed of 19–24 nucleotides (Sessa et al., 2023; Mooren et al., 2014). Their biogenesis is a multi-step process beginning in the cell nucleus, where miRNA-encoding genes are transcribed to produce long primary transcripts known as pri-miRNAs (Polakovičová et al., 2016; Muhuri, 2013). The pri-miRNA is subsequently cleaved by the Drosha-DGCR8 microprocessor complex into a shorter, hairpin-shaped precursor called a pre-miRNA (Romaine et al., 2015; Li, 2021). This pre-miRNA is then transported from the nucleus to the cytoplasm by the protein exportin-5 (Wu et al., 2018). In the cytoplasm, another RNase III enzyme, Dicer, further cleaves the pre-miRNA to produce a mature, double-stranded miRNA duplex (Gao et al., 2023; Johanson et al., 2013).

**FIGURE 1:**
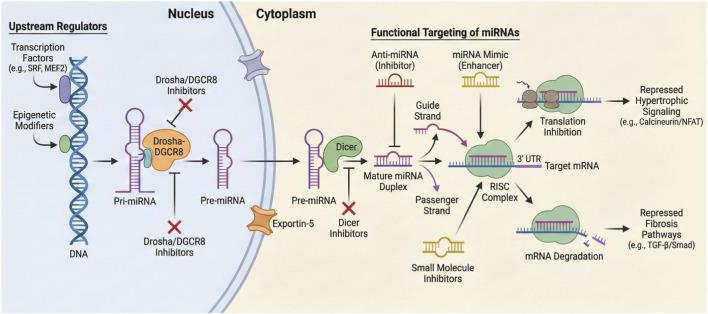
The canonical biogenesis and functional targeting of miRNAs. The schematic illustrates the stepwise maturation of microRNAs, beginning in the nucleus where transcription factors (e.g., SRF, MEF2) and epigenetic modifiers drive the expression of pri-miRNA. The Drosha-DGCR8 complex cleaves this precursor into pre-miRNA, which is subsequently transported to the cytoplasm by Exportin-5. There, Dicer processes the pre-miRNA into a mature duplex, allowing the functional guide strand to load into the RNA-induced silencing complex (RISC). This complex binds to complementary sequences on target mRNAs to induce translation inhibition or degradation, ultimately acting to repress downstream hypertrophic signaling (e.g., Calcineurin/NFAT) and fibrosis pathways (e.g., TGF-β/Smad).

A single strand of this mature duplex is then loaded into a multi-protein complex known as the RNA-induced silencing complex (RISC), which uses the miRNA as a template to identify and bind to its target messenger RNA (mRNA) (Gao et al., 2023; Roberts, 2015). The binding of the RISC-miRNA complex to the 3′-untranslated region (UTR) or, in some cases, noncanonical target sites, of the target mRNA leads to either mRNA degradation or the inhibition of protein translation (Gao et al., 2023; Roberts, 2015; Wilczynska and Bushell, 2015). This post-transcriptional regulatory mechanism is complex and highly interconnected (Ivanov and Anderson, 2013). A single miRNA can regulate the expression of multiple genes, and conversely, a single gene can be regulated by several different miRNAs, often co-regulating an entire biological pathway (Polakovičová et al., 2016; Le et al., 2015). This network-like regulation makes miRNAs powerful agents in controlling cellular processes and maintaining homeostasis (Gao et al., 2023; Demongeot et al., 2013) (Figure 1).

### 2.2 Inter-organ crosstalk: the heart and the exerkine hypothesis

The organism functions as a complex network of interconnected organs and systems, and exercise induces a systemic response that involves intricate communication between them (Luo et al., 2024; James, 2025). This communication extends far beyond direct neural or hormonal signals and is now understood to be mediated by a new class of humoral factors known as “exerkines” (Magliulo et al., 2022). Exerkines are signaling molecules—including peptides, metabolites, and RNA—that are released from metabolically active tissues in response to acute or chronic exercise (Jin et al., 2024; Chow et al., 2022).

The concept of exerkines highlights the emerging understanding of skeletal muscle as a vital endocrine organ (Luo et al., 2024; Chow et al., 2022). Upon contraction, skeletal muscle releases a variety of these bioactive factors, some of which are encapsulated within small membrane-bound vesicles called exosomes (Luo et al., 2024; Rome et al., 2019). Extracellular vesicles (EVs), which include exosomes, serve as a fundamental mechanism for long-range, intercellular and inter-organ communication (Lin et al., 2024; Ch et al., 2025). They are released into the bloodstream and can reach distant organs, where they transfer their cargo of proteins, lipids, and nucleic acids to recipient cells, influencing their physiological state (Luo et al., 2024; Chimal-Vega et al., 2025).

Within this framework, exosomes provide a concrete mechanistic link between physical activity and cardioprotection (Han et al., 2020). The evidence establishes that skeletal muscle releases exosomes in response to exercise (Luo et al., 2024; Vechetti et al., 2021). It is also known that these exosomes can transport their miRNA cargo from the donor cell to a recipient cell, thereby influencing gene expression at a distance (Luo et al., 2024; Schwarzenbach and Gahan, 2019). The heart, as a key target of the systemic effects of exercise, can take up muscle-derived exosomes, which then deliver their miRNA payload (Gabisonia et al., 2022; Han et al., 2020). This process provides a clear and direct pathway through which the biochemical effects of muscle contraction can be translated into beneficial, adaptive gene expression changes within the myocardium (Clerk et al., 2007). In essence, exercise-induced muscle activity orchestrates a humoral signaling cascade, mediated by exosomal miRNAs, that directly confers cardioprotective benefits, positioning skeletal muscle not merely as the power source for locomotion but as a crucial systemic regulator of cardiovascular health.

## 3 Key MicroRNA signatures in cardiac adaptation

### 3.1 Physiological vs. pathological remodeling: the “Athlete’s heart”

The heart’s response to stress is complex and can lead to two distinct forms of left ventricular (LV) hypertrophy (Fernandes et al., 2015; Grossman et al., 1975; Shah et al., 2021). Pathological hypertrophy, often triggered by hypertension or myocardial infarction, is a hallmark of HF and is characterized by ventricular dysfunction, fibrosis, and a disproportionate increase in muscle mass relative to angiogenesis (Fernandes et al., 2015; Shah et al., 2021). In stark contrast, physiological hypertrophy, exemplified by the “athlete’s heart,” is a beneficial adaptation to chronic exercise (Fernandes et al., 2015; La Gerche et al., 2022). This form of remodeling is characterized by preserved or enhanced LV function, increased cardiac muscle mass, augmented angiogenesis, and a lack of fibrosis (Fernandes et al., 2015; Kruszewska et al., 2022). Distinguishing between these two conditions is a major clinical challenge, and miRNAs are emerging as promising molecular markers that may facilitate this critical differential diagnosis (Soplinska et al., 2020; Ho et al., 2022).

### 3.2 The canonical MyomiRs: miR-1 and miR-133

MicroRNA-1 and microRNA-133 are two of the most well-studied muscle-enriched miRNAs, or “myomiRs,” due to their central roles in the development and homeostasis of both skeletal and cardiac muscle (Mitchelson and Qin, 2015; Al-Kafaji et al., 2021). They are co-transcribed and act as cooperative regulatory factors, influencing a wide range of cardiovascular processes (Al-Kafaji et al., 2021).

In models of pathological cardiac hypertrophy, such as those induced by transverse aortic constriction (TAC), miR-1 is consistently downregulated (Melman et al., 2014; Li J. et al., 2022). Preclinical studies have shown that the targeted delivery of a miR-1 mimic to TAC-treated mice can reverse hypertrophy and ventricular dysfunction, with a concomitant reduction in fibrosis (Melman et al., 2014; Karakikes et al., 2013). The anti-hypertrophic effects of miR-1 are mediated by its ability to inhibit a number of genes known to be involved in cardiac hypertrophy, including MEF2a, GATA4, and calmodulin (Melman et al., 2014; Wehbe et al., 2019; Ikeda et al., 2009). Similarly, miR-133 is also downregulated in myocardial hypertrophy, and its overexpression can synergistically inhibit this condition when acting in concert with miR-1 (Liu and Olson, 2010). Beyond hypertrophy, these two miRNAs are implicated in cardiac reprogramming, as their combined injection into ischemic myocardial tissue can transform fibroblasts into cardiomyocyte-like cells, enhancing cardiac function and improving survival (Hodgkinson et al., 2015; Jayawardena et al., 2012).

However, the role of miR-1 in cardiac function is not without complexity. While it appears to be protective against pathological hypertrophy, other studies have documented that the overexpression of miR-1 in rats with ischemic heart disease can exacerbate arrhythmogenesis (Liu et al., 2017; Kura et al., 2020; Mishra et al., 2009). This pro-arrhythmic effect is thought to be mediated by the miRNA’s influence on genes related to potassium channels (e.g., KCNJ2, GJA1) and connexin-43 (Cx43) (Kura et al., 2020; Hancox et al., 2023). This duality suggests that the function of miR-1 is highly context-dependent. Its beneficial anti-hypertrophic effects, which are mediated by targeting transcription factors, may be distinct from its potentially deleterious pro-arrhythmic effects, which involve the regulation of ion channels and gap junctions. The physiological stress of exercise may selectively activate cardioprotective pathways involving miR-1 while mitigating the activation of arrhythmogenic ones, whereas a pathological stressor like ischemia may trigger a different, less favorable signaling cascade. This highlights a critical challenge for therapeutic application and underscores the nuanced nature of miRNA regulation in health versus disease (Quindry and Hamilton, 2013; Wei et al., 2025).

While rodent models consistently show miR-1 downregulation in hypertrophy, human data remains conflicting. Several clinical studies in heart failure patients have reported *elevated* circulating miR-1 levels, potentially reflecting ongoing cardiomyocyte death rather than adaptive downregulation. This discrepancy underscores the challenge of extrapolating tissue-level mechanisms from plasma biomarkers in heterogeneous human populations.

### 3.3 Cardiac-specific regulators: miR-208a and miR-499

Other miRNAs also play highly specific roles in cardiac function and its adaptation to exercise (Kolodziej et al., 2022). MicroRNA-208a is a cardiac-specific miRNA encoded by an intron in the myosin heavy chain gene family (Huang et al., 2021; Callis et al., 2009). In contrast to miR-1, miR-208a is a pro-hypertrophic regulator, and its transgenic expression is sufficient to induce hypertrophic growth of cardiomyocytes (Huang et al., 2021; Nagalingam et al., 2014). It regulates genes involved in the expression of fast- and slow-twitch myofiber proteins and is a key factor in the pathological “myosin switching” that occurs in heart disease (Huang et al., 2021; van Rooij et al., 2009). Significantly, the genetic deletion or therapeutic inhibition of miR-208a in animal models has been shown to attenuate stress-induced cardiac hypertrophy and remodeling, while improving cardiac function and survival (Huang et al., 2021; Montgomery et al., 2011). This makes miR-208a potent and validated therapeutic target for HF (Montgomery et al., 2011; Eding et al., 2017).

Another muscle-enriched miRNA, microRNA-499, has emerged as a promising diagnostic biomarker for cardiac injury (Xin et al., 2016; Velu et al., 2018). Its circulating levels are significantly elevated in patients with acute myocardial infarction (AMI), and this increase is detectable earlier than traditional biomarkers like cardiac troponins (Xin et al., 2016; Aydin et al., 2019; Babuin and Jaffe, 2005). The concentration of circulating miR-499 correlates with the severity of coronary artery disease (Xin et al., 2016; Fazmin et al., 2020). The fact that two closely related myomiRs, miR-208a and miR-499, can serve as a potent therapeutic target and a sensitive diagnostic marker, respectively, demonstrates the central importance of this class of molecules in clinical cardiology (Pinchi et al., 2019; Pourrajab et al., 2016). The utility of miR-499 as a diagnostic tool may be a direct result of the exosome-mediated inter-organ communication discussed previously, as miR-499 is expressed in skeletal muscle and is upregulated in response to ischemic injury (Cheng et al., 2025; Li et al., 2025). This links the biomarker concept directly back to the humoral signaling pathway, creating a cohesive and compelling narrative (Ma et al., 2024) (Table 1).

**TABLE 1:** Key miRNAs and their roles in exercise-induced cardiac adaptation.

miRNA	Tissue of origin	Exercise responsiveness	Validated targets	Functional outcome	References
miR-1	Heart, Muscle	↑Acute (Plasma) ↔ Chronic (Resting)	MEF2a, GATA4, calmodulin	Anti-hypertrophic; modulates arrhythmia risk	Mooren et al. (2014)
miR-133a	Heart, Muscle	↑Acute (Plasma)	Caspase-9, HDAC4	Anti-apoptotic; inhibits fibrosis	Mooren et al. (2014)
miR-208a	Heart (specific)	↓Chronic (Therapeutic goal)	SOX6, MED13, Myostatin	Pro-hypertrophic; drives myosin switching	Alavi-Moghaddam et al. (2018)
miR-499	Heart, Muscle	↑Acute (Plasma)	Calcineurin	Marker of cardiac stress/injury; regulates hypertrophy	Mooren et al. (2014)
miR-21	Fibroblasts, Heart	↑Chronic	PTEN, SPRY2	Anti-fibrotic (context-dependent); pro-angiogenic	Gao et al. (2023)
miR-126	Endothelium	↑Endurance/HIIT	SPRED1, PIK3R2	Promotes angiogenesis and vascular integrity	Gao et al. (2023)
miR-29a	Fibroblasts	↑Chronic	Collagen (COL1A1, COL3A1)	Anti-fibrotic; reduces stiffening	Fernandes et al. (2015)
miR-210	Hypoxic tissue	↑Resistance/HIIT	ISCU, COX10	Improves mitochondrial metabolism in hypoxia	Bye et al. (2013)

### 3.4 Context-dependent duality: the ‘double-edged sword'

It is critical to acknowledge that miRNAs often exert contradictory effects depending on the physiological context. miR-1, for example, is potently anti-hypertrophic in pressure overload models (beneficial) but can be pro-arrhythmic in ischemic conditions by targeting connexin-43 and potassium channels (detrimental) (Kura et al., 2020; Hancox et al., 2023). Similarly, miR-21 is widely recognized as anti-apoptotic (pro-survival) in acute ischemia via the PTEN/Akt pathway, yet in chronic heart failure, it drives pathological fibrosis by activating fibroblasts (TGF-β signaling) (Gao et al., 2023). This duality necessitates precise, context-specific therapeutic targeting rather than broad inhibition or overexpression.

### 3.5 Distinct ncRNA landscapes: HFrEF vs. HFpEF and etiology

It is critical to differentiate between HF phenotypes, as they exhibit distinct ncRNA signatures.

- HFrEF (Systolic Failure): Characterized by cardiomyocyte death and eccentric hypertrophy. Key drivers include miR-1 and miR-133a (downregulated) and pro-hypertrophic miR-208a (upregulated).
- HFpEF (Diastolic Failure): Driven by fibrosis and systemic inflammation. This phenotype is more closely associated with miR-21 (fibrosis), miR-29 (ECM remodeling), and inflammatory circulating miRNAs (e.g., miR-146a).
- Ischemic Etiology: Ischemic HF specifically upregulates hypoxia-sensitive miRNAs like miR-210 and angiogenic miR-126, distinct from the pressure-overload profiles seen in hypertensive HF (Sanchis-Gomar et al., 2021) (144–146).

## 4 Exercise modality and intensity: differential miRNA expression

The adaptive response of the cardiovascular system to physical activity is not uniform; it is highly dependent on the type and intensity of the exercise performed (Liu et al., 2017; Ma et al., 2024). Endurance training, characterized by prolonged, high-dynamic activity like running or cycling, induces eccentric hypertrophy, leading to an increase in left ventricular chamber size and capacity (Fernandes et al., 2015; Bletsa et al., 2023). In contrast, resistance training, which involves high-power loads, typically results in concentric hypertrophy, characterized by increased ventricular wall thickness (Liu et al., 2017; Barauna et al., 2007). These distinct physiological adaptations are mirrored by differential miRNA expression profiles, suggesting a highly specific molecular response to different mechanical stresses (Kirby and McCarthy, 2013).

Studies have documented that a single bout of acute endurance exercise, such as a marathon, leads to an immediate increase in circulating miRNAs, including miR-499 and miR-208b (Soplinska et al., 2020). However, the levels of some of these miRNAs, such as miR-499, return to pre-exercise baseline within 24 h (Mooren et al., 2014). Other miRNAs, such as miR-1 and miR-133a, have been shown to correlate positively with aerobic performance parameters like maximal oxygen uptake (VO2​max) and running speed (Bye et al., 2013; Mooren et al., 2014). These findings underscore the transient and sustained changes that occur in the miRNA landscape. For example, while acute exercise may lead to a transient increase in miR-133a, long-term training may result in no significant change, which highlights the importance of studying both acute and chronic effects.

**FIGURE 2:**
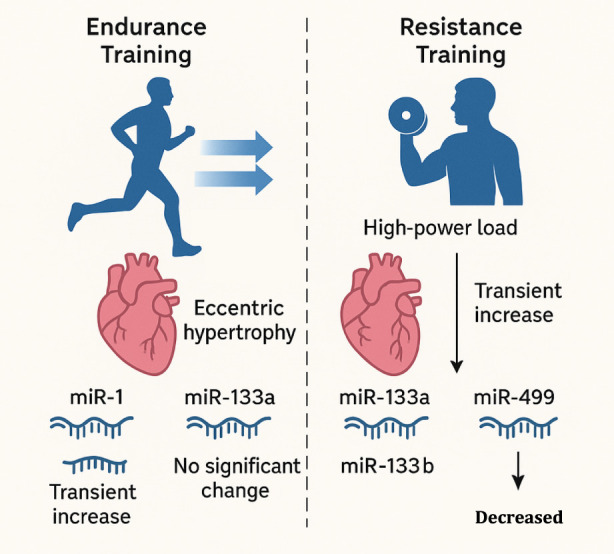
Differential miRNA expression in response to exercise. Endurance training (aerobic activity) induces eccentric hypertrophy and transient increases in circulating miRNAs such as miR-1 and miR-133a. Resistance training (high-power load) leads to concentric hypertrophy, with decreases in muscle miR-133b and variable increases in plasma miR-133a and miR-499, correlating with strength adaptation.

Similarly, resistance training elicits its own unique miRNA signature. In a study on older adults, resistance training caused a decrease in muscle tissue miR-133b, while plasma levels of multiple miRNAs, including miR-133a and miR-499, tended to increase (Zhang et al., 2015; Ultimo et al., 2018). The changes in these miRNAs were strongly correlated with improvements in muscle strength (Zhang et al., 2015). Recent evidence highlights distinct signatures for High-Intensity Interval Training (HIIT) compared to moderate-intensity continuous training (MICT). For instance, HIIT has been shown to induce a more rapid and robust upregulation of angiogenic miRNAs, such as miR-126 and miR-16, likely driven by the higher shear stress and hypoxic stimulus associated with interval peaks (Cui et al., 2017). Conversely, resistance training preferentially mobilizes mechano-sensitive miRNAs (e.g., miR-133a, miR-486) involved in PI3K/Akt/mTOR signaling to support concentric hypertrophy and protein synthesis (Zhang et al., 2015; Ultimo et al., 2018). Understanding these modality-specific profiles is crucial for prescribing ‘precision exercise' as a therapeutic adjuvant. A comparative summary of the distinct ncRNA signatures elicited by aerobic, resistance, and high-intensity interval training is provided in Table 2. This evidence demonstrates that miRNA expression is not a monolithic response but rather a sophisticated, dynamic regulatory network that is fine-tuned based on the precise nature of the physical stress (Pu et al., 2019). To fully comprehend the role of miRNAs in cardioprotection, it is necessary to consider the time- and intensity-dependent nature of their expression (Aghabozorg Afjeh and Ghaderian, 2013). This dynamic interplay has significant implications for both the development of precise biomarkers and the design of therapeutic interventions, as the “correct” miRNA signature may be unique to an endurance athlete versus a strength athlete, or for acute recovery versus chronic adaptation (Pickering and Kiely, 2019) (Figure 2). The divergence in ncRNA profiles between exercise modalities is likely driven by distinct upstream mechanical stimuli. Endurance training (volume overload) significantly increases laminar shear stress on the endothelium, triggering the release of flow-sensitive miRNAs like miR-126 via the KLF2 transcription factor pathway. Conversely, Resistance training (pressure overload) generates high mechanical tension on the sarcolemma, activating mechanosensitive channels that preferentially mobilize muscle-enriched miRNAs (myomiRs) like miR-133a and miR-486 to regulate sarcomere assembly and protein synthesis (Zhang et al., 2015; Cui et al., 2017). This suggests that exercise prescriptions can be tailored to target specific molecular pathways: high-shear aerobic intervals for vascular adaptation, and high-load resistance training for myocardial hypertrophy.

**TABLE 2:** Differential ncRNA responses by exercise modality.

Exercise modality	Physiological stimulus	Key ncRNA response	Functional adaptation	References
Aerobic/Endurance	Volume overload, Shear stress	**↑miR-1, miR-133a** (acute) **↑miR-126, miR-29a** (chronic)	Eccentric hypertrophy, Angiogenesis, Anti-fibrosis	Fernandes et al. (2015); Qian et al. (2024); Sessa et al. (2023); Mooren et al. (2014); Uhlemann et al. (2014)
Resistance Training	Pressure overload, Mechanical tension	**↓miR-133b** (muscle) **↑miR-486**	Concentric hypertrophy, Myofibrillar protein synthesis	Zhang et al. (2015); Ultimo et al. (2018)
HIIT	Hypoxia, Metabolic stress	**↑miR-21, miR-222** **↑miR-499** (rapid release)	Mitochondrial biogenesis, Rapid metabolic switching	Fan and Evans. (2017); Mooren et al. (2014)
Marathon (Acute)	Extreme metabolic/oxidative stress	**↑miR-208b, miR-499** (transient) **↑Inflammation-related miRNAs**	Transient cardiomyocyte stress/injury signal	Soplinska et al. (2020); Ho et al. (2022)

Bold values indicate miRNAs showing statistically significant changes in expression (typically p < 0.05) relative to baseline or sedentary controls, as reported in the cited studies.

### 4.1 Sex, Age, and training status: modulators of miRNA responsea

Individual biological variables significantly influence the miRNA response to exercise, acting as ‘effect modifiers' on the cardioprotective landscape. Sex Differences: Hormonal regulation plays a pivotal role; for instance, estrogen modulates the biogenesis of angiogenesis-regulating miRNAs. Males often exhibit a more robust upregulation of miR-126 and miR-21 following high-intensity interval training (HIIT), promoting vascular adaptation, whereas females may show distinct profiles favoring anti-inflammatory pathways like miR-223 (Hiam et al., 2023). Impact of Aging: Aging is associated with a generalized downregulation of plasticity-related miRNAs (e.g., miR-486, miR-146a), leading to a ‘blunted' response to exercise stimuli. Older adults often require higher intensity or longer duration training to elicit the same miRNA-mediated anabolic and anti-inflammatory signals seen in younger cohorts, contributing to sarcopenia and delayed recovery (Drummond et al., 2008; Olivieri et al., 2013). Training Status: Long-term training alters the baseline ‘set-point.' Elite athletes exhibit elevated basal levels of cardioprotective miR-1 and miR-133a, and their acute response to exercise is often rapid and transient—indicative of an efficient adaptive system—whereas sedentary individuals display prolonged, stress-related elevations in circulating miRNAs (e.g., miR-499) (Baggish et al., 2011; Sawada et al., 2013) (Table 3).

**TABLE 3:** Sex, age, and training status as modulators of miRNA response to exercise.

Factor	miRNA	Baseline expression differences	Response to exercise	Mechanism/Implications	References
Sex (Males vs. Females)	miR-21, miR-222	Higher increase in males at rest; distinct profiles in skeletal muscle	Significant increase in males post-acute endurance; no change in females	Regulates cardiovascular adaptation via endothelial function and angiogenesis; hormonal influences (estrogen/testosterone) modulate biogenesis	Denham et al. (1985)
Sex (Males vs. Females)	miR-126	More pronounced in males post-HIIT	Increased in young trained males; blunted in females	Involved in vascular health; sex-biased regulation affects metabolic and inflammatory pathways	Cui et al. (2017)
Sex (Males vs. Females)	miR-223	Female-specific elevation	Modulates immune/inflammatory responses post-exercise	Estrogen modulation; influences muscle regeneration and inflammation	Mooren et al. (2014)
Sex (Males vs. Females)	miR-1, miR-133a	Elevated in endurance-trained males	Differential in PBMCs during maximal exercise; higher in males for immune regulation	Hormonal (testosterone) enhancement of hypertrophy pathways; sex-specific muscle fiber composition	Cheema et al. (2020), Baggish et al. (2011)
Sex (Males vs. Females)	miR-146a	Sex-biased in muscle tissues	Altered in distance running; unique in females for pathologic states	Epigenetic factors; affects recovery/adaptation via inflammation control	Hicks et al. (2018)
Age (Young vs. Older)	miR-486	Downregulated with age	Blunted response in older adults to resistance exercise	Contributes to sarcopenia; impairs muscle growth/repair	Drummond et al. (2008)
Age (Young vs. Older)	miR-21, miR-146a	Declined in older adults	Less pronounced restoration via exercise in elderly	Linked to impaired inflammatory resolution; increased muscle damage susceptibility	Olivieri et al. (2013); Margolis et al. (2017)
Age (Young vs. Older)	miR-92a	Downregulated in youth post-aerobic	Differential biomarkers for gait speed; varied mediation of adaptations	Reduced biogenesis efficiency; oxidative stress alters responsiveness	Margolis et al. (2017)
Age (Young vs. Older)	Bone-regulating miRNAs (e.g., miR-125b)	Age-dependent decline	Varied response to resistance; systemic effects on bone-muscle axis	Hormonal changes; impacts anabolic capacity and regenerative potential	Margolis et al. (2017)
Training Status (Trained vs. Untrained)	miR-1, miR-133a	Elevated in trained athletes at baseline	Rapid return to baseline in trained; prolonged in untrained	Reflects muscle differentiation/growth; enhanced recovery mechanisms	Nielsen et al. (2013) Baggish et al. (2011)
Training Status (Trained vs. Untrained)	miR-20a-5p	Altered in whole blood post-training	Provides cardiovascular protection; downregulates PTEN	Improves mitochondrial function; antioxidant capacity in trained	Uhlemann et al. (2014)
Training Status (Trained vs. Untrained)	miR-126	More pronounced in trained	Transient changes; faster normalization in trained	Vascular health; indicative of efficient adaptation processes	Sawada et al. (2013)
Interactions (Sex + Age + Training)	miR-126	Pronounced in young trained males vs. older sedentary females	Combined effects on vascular responses	Hormonal/aging/physical history interplay; personalized prescription needs	Margolis et al. (2017); Cui et al. (2017)
Interactions (Sex + Age + Training)	Salivary miRNAs (e.g., miR-223)	Sex-specific with age intersections	Responses to endurance; intergenerational via sperm miRNAs	Integrated models for multifaceted adaptations	Hicks et al. (2018)

## 5 Translational potential: miRNAs as biomarkers and therapeutics

### 5.1 Circulating miRNAs as non-invasive biomarkers

The remarkable stability of miRNAs in circulation, both as free molecules and within exosomes, makes them ideal candidates for non-invasive biomarkers of cardiovascular health (Bye et al., 2013; Khalyfa and Gozal, 2014). There is a critical clinical need for new biomarkers to differentiate the benign, physiological “athlete’s heart” from pathological conditions like hypertrophic cardiomyopathy, a distinction that is often challenging with conventional diagnostic methods (Soplinska et al., 2020; Eijsvogels et al., 2016). miRNAs offer a promising solution.

Studies have found that circulating levels of miRNAs, such as miR-21, miR-210, and miR-222, are increased in healthy individuals with low maximal oxygen uptake (VO2​max) (Bye et al., 2013; Eijsvogels et al., 2016). Notably, these levels were not correlated with traditional cardiovascular risk factors like blood pressure or cholesterol, suggesting they may serve as new, independent markers of fitness level and future CVD risk (Bye et al., 2013; Eijsvogels et al., 2016). Other research indicates that changes in circulating miRNAs following an acute bout of exercise, such as a marathon, correlate with cardiac injury markers like troponin and creatine kinase-MB (Soplinska et al., 2020). These findings suggest that miRNAs can provide valuable insights into cardiac adaptation processes and may help facilitate the differential diagnosis between adaptive changes and pathology in athletes (Soplinska et al., 2020; Eijsvogels et al., 2016).

It is critical to distinguish between miRNAs that actively drive cardioprotection and those that are merely ‘passenger' biomarkers of physiological stress. For instance, while circulating miR-499 levels correlate with exercise intensity, they primarily reflect transient cardiomyocyte membrane permeability rather than a regulatory signal (Soplinska et al., 2020). In contrast, exosomal miR-342-5p has been proven via gain-of-function studies to actively suppress apoptotic signaling in recipient cardiomyocytes (Hou et al., 2019). Future biomarker research must move beyond correlation to validate functional biological activity.

Despite their potential, the clinical utility of circulating miRNAs is hampered by technical variability.

1. Normalization: There is no universal endogenous control for plasma miRNAs (like GAPDH for tissue). Using *C. elegans* spike-ins (cel-miR-39) corrects for extraction efficiency but not for biological variations like plasma volume or hemolysis.
2. Diurnal Variation: Circulating levels of specific miRNAs (e.g., miR-16) oscillate with circadian rhythms, necessitating standardized sampling times (e.g., morning fasting).
3. Reproducibility: Differences in extraction kits and detection platforms (qPCR vs. localized hybridization) contribute to poor inter-study reproducibility. Establishing robust reference intervals accounting for age, sex, and ethnicity is a prerequisite for clinical adoption.

### 5.2 miRNA-based therapeutic strategies

The identification of miRNAs that mediate cardioprotective effects has paved the way for a new class of pharmacological interventions. The two main therapeutic approaches involve either administering synthetic miRNA mimics to restore a downregulated, cardioprotective miRNA or using antagonists (antagomiRs) to inhibit a detrimental miRNA (Romaine et al., 2015). The concept of targeting miRNAs *in vivo* has been demonstrated as feasible in both animal and clinical studies, with synthetic antagonists already in Phase II clinical trials for other conditions (Liu et al., 2017; Chakraborty et al., 2021).

#### 5.2.1 Establishing causality: gain- and loss-of-function studies

While many studies report associative changes in ncRNA levels post-exercise, establishing them as *bona fide* mediators of cardioprotection requires rigorous gain- and loss-of-function experiments. For example, the specific knockdown of miR-208a using antisense oligonucleotides (antagomiRs) in hypertensive rats was sufficient to prevent pathological remodeling, proving its causal role in the phenotype (Montgomery et al., 2011). Similarly, the therapeutic injection of miR-1 mimics into ischemic hearts directly repressed hypertrophic genes (e.g., *GATA4*, *MEF2a*), causally linking miR-1 restoration to functional recovery (Melman et al., 2014; Karakikes et al., 2013). In the context of exosomes, causality has been demonstrated by transferring exosomes from exercised mice into sedentary myocardial infarction models; this transfer conferred protection that was abolished when specific cargo, such as miR-342-5p, was depleted, confirming the miRNA as the active ingredient (Hou et al., 2019). Future research must prioritize such mechanistic validation over descriptive profiling.

#### 5.2.2 Safety and regulatory hurdles

Despite the promise of miRNA therapeutics, significant safety barriers impede clinical translation.

1. Off-Target Effects: Since a single miRNA targets hundreds of mRNAs, mimics may inadvertently repress tumor suppressors or essential metabolic genes in non-cardiac tissues.
2. Delivery Toxicity: Viral vectors (AAV) carry risks of immunogenicity and genomic integration, while lipid nanoparticles (LNPs) often accumulate in the liver and spleen, limiting cardiac uptake.
3. Regulatory Complexity: Regulatory agencies like the FDA require stringent evaluation of potential ‘on-target, off-tissue' toxicities. The theoretical risk of inducing arrhythmias (e.g., via miR-1 overexpression) or fibrosis (via miR-21 inhibition) necessitates rigorous, long-term safety data in large animal models before human trials can proceed (Liu et al., 2017; Chakraborty et al., 2021).

The most compelling example of this therapeutic potential is the inhibition of miR-208a (Caroli et al., 2013). As a key driver of pathological hypertrophy, miR-208a is a prime target for intervention. Preclinical studies have shown that the systemic delivery of an antisense oligonucleotide, antimiR-208a, to rats with hypertension-induced HF prevented pathological myosin switching, reversed cardiac remodeling, and significantly improved both cardiac function and survival (Montgomery et al., 2011). The success of this approach validates the potential of oligonucleotide-based therapies for modulating cardiac miRNAs and offers a precise strategy for treating heart disease (Montgomery et al., 2011; Baumann and Winkler, 2014).

**FIGURE 3:**
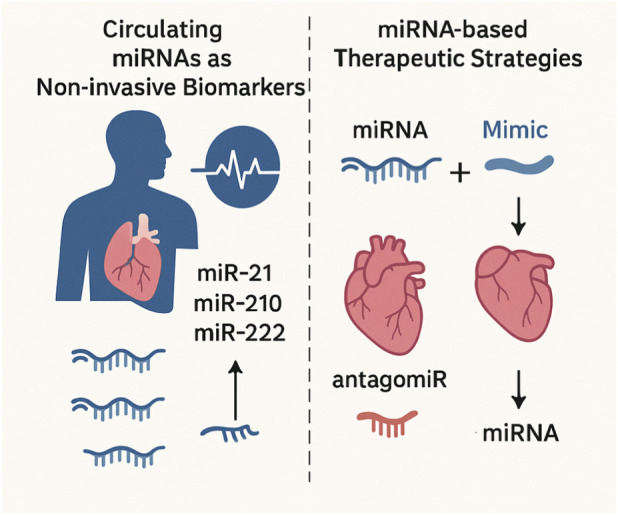
Circulating miRNAs and therapeutic strategies: Circulating miRNAs (e.g., miR-21, miR-210, miR-222) act as non-invasive biomarkers of cardiovascular health and adaptation. Therapeutic approaches include miRNA mimics to restore cardioprotective miRNAs and antagomiRs to inhibit detrimental ones (e.g., miR-208a), offering targeted strategies against pathological hypertrophy.

This research is leading to the development of what can be described as “exercise mimetics” (Fan and Evans, 2017). The snippets identify specific miRNAs that mediate the beneficial effects of exercise on the heart, such as miR-1, miR-133, miR-29a, and miR-126 (Fernandes et al., 2015; Wang et al., 2018; Li and Hallajzadeh, 2025). The existence of synthetic miRNA mimics and antagonists allows for the direct manipulation of these molecular pathways. Therefore, these miRNA-based therapies could potentially provide the salutary effects of physical activity at a molecular level for patients who are unable to exercise (Jin et al., 2024; Ishida and Selaru, 2013; Long and Danesh, 2022). This positions the current research not merely as an academic study of a biological mechanism but as the foundational work for a new, and potentially more precise, class of pharmacological interventions that can replicate the therapeutic benefits of exercise to combat the global burden of CVD (Hagen et al., 2025; Malm et al., 2022) (Figure 3).

### 5.3 Current clinical investigations

While exercise-specific miRNA trials are primarily observational, direct therapeutic targeting has reached clinical stages.

- Therapeutic Targeting (NCT04045405): The Phase 1b trial of CDR132L, a synthetic antisense oligonucleotide inhibitor of miR-132, demonstrated safety and efficacy in patients with ischemic heart failure. By inhibiting the pathological hypertrophy driver miR-132, this drug mimics the anti-remodeling effects typically induced by chronic training.
- Biomarker Validation (The HUNT Study): Large-scale observational cohorts such as the HUNT Study () have clinically validated circulating miR-210 and miR-222 as robust predictors of low VO2max, establishing them as surrogate markers for exercise capacity in the general population.
- Future Interventions: Emerging protocols are now focusing on monitoring exosomal miRNA signatures (e.g., miR-126, miR-146a) to predict responsiveness to cardiac rehabilitation, distinguishing ‘responders' from ‘non-responders' in heart failure cohorts.

## 6 Long non-coding RNA–microRNA interactions in exercise-induced cardioprotection

While miRNAs function primarily as post-transcriptional repressors, Long Non-Coding RNAs (lncRNAs) operate through more diverse and complex mechanisms. Beyond acting as ‘sponges' for miRNAs (the competing endogenous RNA or ceRNA hypothesis), lncRNAs serve as chromatin scaffolds, transcriptional co-activators, and guides for epigenetic modifiers (e.g., EZH2). In the context of the exercising heart, this allows lncRNAs to orchestrate broad gene programs—switching entire metabolic or fibrotic networks on or off—rather than fine-tuning single targets as miRNAs often do (Hu et al., 2021).

Exercise-induced cardioprotection involves molecular adaptations that mitigate cardiovascular damage, with long non-coding RNAs (lncRNAs) and microRNAs (miRNAs) playing interactive roles in regulating cardiac remodeling, inflammation, and mitochondrial function. In rat models of chronic HF, aerobic exercise inhibits lncRNA MALAT1 expression, improving cardiac function through interactions with miR-150-5p and modulation of the PI3K/Akt pathway (Hu et al., 2021). Similarly, endurance exercise alters lncRNAs H19, GAS5, and MIAT in myocardial infarction rat hearts, influencing fibrosis and remodeling via miRNA sponging (Farsangi et al., 2021). In exosomes from long-term exercise, lncRNA CRNDE sponges miR-489-3p to target Nrf2, reducing apoptosis and oxidative stress in hypoxic cardiomyocytes (Han et al., 2024). These interactions highlight how exercise modulates ncRNA networks to enhance cardiac resilience.

In diabetic cardiomyopathy models, exercise downregulates mitochondrial gene Pdk4 through a network of 144 lncRNAs interacting with five miRNAs: miR-138-5p, miR-149-3p, miR-484, miR-3084b-3p, and miR-6323 (Wang et al., 2024). Specifically, 59 lncRNAs interact with miR-484, which targets Yap1 to modulate cardiomyocyte viability and inflammation, shifting metabolism from fatty acid oxidation to glucose utilization (Wang et al., 2024). In myocardial infarction settings, lncRNA H19 acts as a ceRNA for miR-675-5p, promoting angiogenesis and inhibiting apoptosis via miR-103/107 suppression and FADD enhancement (Huang et al., 2020; Gong et al., 2017; Zhou et al., 2018). Exercise upregulates H19 post-infarction, activating autophagy through miR-139/Sox8 signaling (Gong et al., 2017).

High-intensity exercise upregulates lncRNA GAS5 in infarction models, regulating the miR-217/SIRT1 pathway to attenuate remodeling (Zhang et al., 2024). Moderate exercise suppresses lncRNA MIAT, reducing fibrosis by inhibiting TGF-β and sequestering miR-24 (Qu et al., 2017). Swimming exercise elevates lncRNA Mhrt and Mhrt779, interacting with Brg1 to inhibit pro-hypertrophic genes and modulate HDAC2/AKT/GSK3β pathways (Lin et al., 2021). In obese adolescents, exercise modulates the MALAT1/miR-320a axis to improve endothelial function (Zhao et al., 2021). These examples demonstrate tissue-specific lncRNA-miRNA axes responsive to exercise intensity and duration.

miRNAs alone contribute to cardioprotection, but their interactions with lncRNAs amplify effects. Endurance exercise upregulates miR-126, promoting angiogenesis via VEGF signaling in diabetic rats (Uhlemann et al., 2014; Gaeini and Mo’tamedi, 2016). miR-1 and miR-133 increase during training, regulating apoptosis and hypertrophy genes (Peng et al., 2020). miR-21 modulates remodeling by targeting PTEN in PI3K/AKT pathways (Wang et al., 2024). miR-146a and miR-155 suppress inflammation via NF-κB, reducing TNF-α and IL-6 (McCormack et al., 2023). miR-499 and miR-208a enhance mitochondrial biogenesis by regulating oxidative phosphorylation genes (Yamamoto et al., 2012).

In interaction contexts, lncRNAs serve as ceRNAs. H19 sponges miR-139 to protect H9c2 cells from hypoxia (Gong et al., 2017). GAS5 modulates miR-217 to regulate SIRT1, inhibiting fibrosis (Zhang et al., 2024). CRNDE sponges miR-489-3p, preventing Nrf2 suppression and oxidative stress (Han et al., 2024). CYTOR, upregulated in muscle post-exercise, enhances myogenesis, potentially interacting with miRNAs in cardiac-skeletal crosstalk (Wohlwend et al., 2021). In DCM, lncRNAs sponge miR-138-5p (16 interactions) to regulate infarction and apoptosis via Pdk4 (Wang et al., 2024).

Mechanistically, these interactions involve sponging, where lncRNAs sequester miRNAs, derepressing targets. In exercise, this modulates PI3K/Akt, enhancing survival (MALAT1/miR-150-5p) (Hu et al., 2021). TGF-β inhibition by MIAT/miR-24 reduces fibrosis (Qu et al., 2017). Chromatin remodeling via Mhrt/Brg1 inhibits hypertrophy (Lin et al., 2021). In mitochondria, lncRNA-miRNA-Pdk4 network shifts metabolism, reducing ROS (Wang et al., 2024). Exosomal delivery, as with CRNDE/miR-489-3p, facilitates interorgan protection (Han et al., 2024).

For myocardial infarction, exercise upregulates miR-126-3p, targeting SPRED1/PIK3R2 for angiogenesis (Gaeini and Mo’tamedi, 2016). miR-21a-5p regulates lipid metabolism via FABP7/HMGCR (Wang et al., 2020). miR-133a-5p reduces apoptosis by targeting CASP3/8/9 (Peng et al., 2020). miR-29a-3p inhibits TGF-β1/SMAD2/3, reducing fibrosis (Wang et al., 2018). miR-222-3p promotes growth via HIPK1/P27 (Liu et al., 2009). miR-17-3p targets TIMP-3/PTEN for hypertrophy (Shi et al., 2017). miR-486-5p inhibits PTEN/FOXO1 for IRI protection (Li et al., 2018). miR-342-5p targets CASP9/JNK2, attenuating IRI (Hou et al., 2019).

**FIGURE 4:**
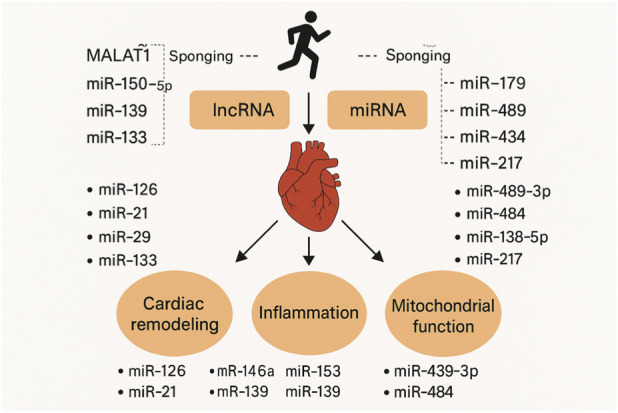
The key long non-coding RNA (lncRNA)–microRNA (miRNA) interaction networks through which exercise induces cardioprotection. Aerobic, endurance, and high-intensity training modulate cardiac and exosomal lncRNAs—including MALAT1, H19, GAS5, MIAT, Mhrt, CRNDE, and CYTOR—leading to altered miRNA availability via competitive endogenous RNA (ceRNA) mechanisms. Exercise-suppressed MALAT1 enhances miR-150-5p activity to activate PI3K/Akt signaling and improve cardiac function in chronic HF. Exercise-induced H19 regulates fibrosis, angiogenesis, and autophagy by sponging miR-139, miR-675-5p, and miR-103/107. GAS5 upregulation during high-intensity training activates the miR-217/SIRT1 axis, reducing post-infarction remodeling. MIAT suppression limits TGF-β–mediated fibrosis through miR-24. Swimming-induced Mhrt and Mhrt779 inhibit Brg1-dependent hypertrophic signaling and modulate HDAC2/AKT/GSK3β pathways. Exosomal CRNDE binds miR-489-3p to derepress Nrf2, decreasing oxidative stress and apoptosis in hypoxic cardiomyocytes.

In HF, exercise restores miR-125b-3p/miR-1290, improving ejection fraction (Sanchis-Gomar et al., 2021). miR-31a-5p/miR-214-3p enhance function in hiPSC-CMs (Plaza-Diaz et al., 2022). In obesity, miR-16-5p/miR-208a-3p downregulation restores VEGF and prevents hypertrophy (Wadley et al., 2019). Exosomal miR-455-5p/miR-29b-3p reduce MMP9 in diabetes Interorgan crosstalk involves exosomal miR-342-5p from heart/BAT, reducing apoptosis (Zhao et al., 2022). Lung-derived miR-421-3p modulates ACE2 post-exposure (Maemura et al., 2018). These networks suggest therapeutic potential, with miR-132-3p/miR-92a-3p targeted in trials (Gallant‐Behm et al., 2018; Täubel et al., 2021). Future research should validate lncRNA-miRNA-Pdk4 in humans (Wang et al., 2024). Explore novel miRNAs like miR-3084b-3p (Wang et al., 2024). Integrate with epigenomics for personalized interventions (Correia et al., 2021; Pereira et al., 2020; Wu et al., 2024). In conclusion, lncRNA-miRNA interactions, such as H19/miR-139 and GAS5/miR-217, mediate exercise cardioprotection by regulating apoptosis, fibrosis, and metabolism (Gong et al., 2017; Zhang et al., 2024). These axes offer biomarkers and targets for CVD management (Figure 4) (Zhang et al., 2020).

Exercise also modifies broader lncRNA–miRNA–mRNA metabolic networks, such as the 144-lncRNA regulatory system targeting Pdk4 in diabetic cardiomyopathy through miR-138-5p, miR-149-3p, miR-484, miR-3084b-3p and miR-6323. Upregulated pro-angiogenic and pro-survival miRNAs—including miR-126, miR-21, miR-133, miR-146a, miR-155, miR-499, and miR-208a—contribute to reduced inflammation, enhanced mitochondrial biogenesis, and cardiomyocyte survival. Collectively, these lncRNA–miRNA axes mediate anti-apoptotic, anti-fibrotic, metabolic, and mitochondrial adaptations that underpin exercise-induced cardioprotection (Table 4). Collectively, these lncRNA–miRNA axes function as molecular switches that translate the mechanical and metabolic stimuli of exercise into stable alterations in cardiac gene expression, primarily by regulating fibrosis and mitochondrial adaptation.

**TABLE 4:** Long non-coding RNA–microRNA interactions in exercise-induced cardioprotection.

lncRNA	miRNA	Interaction type	Exercise context/Model	Cardioprotective effect	Mechanism/Details	References
MALAT1	miR-150-5p	Sponging/Inhibition	Aerobic exercise in chronic HF rats	Improves cardiac function; reduces remodeling	Modulates PI3K/Akt pathway; inhibits expression post-exercise	Hu et al. (2021)
H19	miR-675-5p, miR-103/107, miR-139	ceRNA/Sponging	Endurance exercise in MI rat hearts	Reduces fibrosis; promotes angiogenesis/autophagy	Suppresses FADD; activates Sox8 signaling; upregulated post-infarction	Farsangi et al. (2021); Gong et al. (2017); Zhou et al. (2018)
GAS5	miR-217	Regulation/Sponging	High-intensity exercise in infarction models	Attenuates remodeling; inhibits fibrosis	Regulates SIRT1 pathway; upregulated by exercise	Zhang et al. (2024) Farsangi et al. (2021)
MIAT	miR-24	Sequestration	Moderate exercise in post-infarct	Reduces fibrosis; suppresses TGF-β	Downregulated by exercise; pro-fibrotic inhibition	Qu et al. (2017) Farsangi et al. (2021)
CRNDE	miR-489-3p	Sponging	Long-term exercise exosomes in hypoxic cardiomyocytes	Reduces apoptosis/oxidative stress	Targets Nrf2; exosomal delivery enhances protection	Han et al. (2024)
Mhrt/Mhrt779	N/A (interacts with Brg1)	Chromatin remodeling	Swimming exercise in hypertrophy	Inhibits pro-hypertrophic genes; anti-hypertrophic memory	Modulates HDAC2/AKT/GSK3β; elevated by exercise	Lin et al. (2021)
MALAT1	miR-320a	Axis modulation	Exercise in obese adolescents	Improves endothelial function	Regulates vascular health; modulated in obesity	Zhao et al. (2021)
Multiple (144 lncRNAs)	miR-138-5p, miR-149-3p, miR-484, miR-3084b-3p, miR-6323	Network sponging	Exercise in diabetic cardiomyopathy	Shifts metabolism; downregulates Pdk4	59 lncRNAs with miR-484 target Yap1; reduces fatty acid oxidation	Wang et al. (2024)
CYTOR	Various (potential)	Enhancement	Post-exercise in muscle (cardiac crosstalk)	Promotes myogenesis; potential cardiac benefits	Upregulated in fast-twitch; intersects with miRNA networks	Wohlwend et al. (2021)
H19	miR-139	Sponging	Hypoxia protection in H9c2 cells	Protects against injury; activates autophagy	Derepresses targets for cell survival	Gong et al. (2017)

## 7 Circular RNA–microRNA interactions in exercise-induced cardioprotection

In fetal hearts of pregestational diabetes models, maternal exercise normalizes dysregulated circRNA-miRNA pairs, including circ_0003226 and circ_0015638 interacting with miR-351-5p, as well as circ_0002768 with miR-3102-3p.2-3p (Ye et al., 2023). This regulation involves a network of five circRNAs, 12 miRNAs, and 28 mRNAs, reversing diabetes-induced alterations in 188 circRNAs, 57 miRNAs, and 506 mRNAs. Maternal exercise differentially regulates 188 circRNAs in diabetic pregnancy fetal hearts, compared to 206 circRNAs dysregulated by diabetes alone (Ye et al., 2023). The circRNA-miRNA interactions modulate gene expression relevant to cardiac development, with maternal exercise partially normalizing expression profiles to protect against diabetes-induced cardiac abnormalities (Ye et al., 2023). Specifically, the normalized pairs like circ_0003226/miR-351-5p target mRNAs involved in cardiac morphogenesis, reducing risk of congenital heart defects (Ye et al., 2023).

In myocardial ischemia-reperfusion injury models, circ-RHOJ.1 targets the miR-124-3p/NRG-1 axis to regulate cardiomyocyte proliferation and apoptosis (Xu et al., 2022). circANXA2 inhibits miR-133 expression, promoting myocardial apoptosis (Zong and Wang, 2020). circHIPK3 binds miR-124-3p, aggravating injury (Bai et al., 2019). circ_0050908 sponges miR-324-5p to upregulate TRAF3, worsening reperfusion damage (Ghaffari-Nazari et al., 2022). Mmu-circ-0001380 modulates the miR-106b-5p/Phlpp2 axis, and its knockdown attenuates injury (Wang et al., 2023a). circRNA Fbxl5 sponges miR-146a, regulating cardiomyocyte apoptosis (Li D. et al., 2022). circHDAC9 regulates the miR-671-5p/SOX4 signaling axis in reperfusion (Liu et al., 2024). circPAN3 targets the miR-421/Pink1 axis, suppressing autophagy to ameliorate injury (Zh et al., 2021). circRNA PVT1 targets miR-125b and miR-200a, and its silencing prevents injury in rats (Luo et al., 2021). Exercise training induces miR-20a-5p increase, which interacts with the 3′UTR of PTEN to downregulate its expression, promoting cell survival and proliferation in CVD models (Gao et al., 2023). miR-130a-5p suppresses reperfusion injury by downregulating the HMGB2/NF-κB axis (Zhang et al., 2020). miR-214-3p protects against injury by targeting lysine demethylase 3A (Wugeng et al., 2023). miR-144 acts as a circulating effector of remote ischemic preconditioning (Kohns et al., 2019). FGF21 reduces miR-145-mediated autophagy to protect against reperfusion (Gaeini and Mo’tamedi, 2016). miR-497 inhibition ameliorates anoxia/reoxygenation by suppressing apoptosis and enhancing autophagy (Li et al., 2015). miR-221 upregulation inhibits hypoxia/reoxygenation-induced autophagy via DDIT4/mTORC1 and Tp53inp1/p62 pathways (Chen et al., 2016). miR-638 overexpression attenuates hypoxia/reoxygenation by targeting ATG5 (Hou et al., 2019). miR-142-3p is upregulated by lncRNA TUG1 inhibition to ameliorate injury via HMGB1- and Rac1-induced autophagy (Su et al., 2019). miR-181d-5p is inhibited by circ_ZNF512 to limit autophagy and promote injury (Zh et al., 2021).

Exercise-induced circUtrn is upregulated in swimming-trained adult mouse cardiomyocytes, required for physiological hypertrophy, and prevents myocardial ischemia-reperfusion injury (Kühnisch et al., 2023). *In vivo*, circUtrn deficiency impairs exercise-induced hypertrophy and exacerbates I/R injury in mice (Kühnisch et al., 2023). miR-17-3p contributes to exercise-induced cardiac growth and protects against I/R by targeting TIMP-3/PTEN (Shi et al., 2017). miR-125a-5p delivery improves recovery from I/R (Thum, 2012). miR-30c-5p promotes I/R by activating NF-κB and targeting SIRT1 (Caño-Carrillo et al., 2023). miR-132 promotes oxidative stress-induced pyroptosis by targeting sirtuin 1 (Zhou et al., 2018). miR-29a inhibition protects against I/R by targeting SIRT1 and suppressing oxidative stress and NLRP3-mediated pyroptosis (Ding et al., 2020). miR-22 upregulation contributes to I/R by interfering with mitochondrial function (Du et al., 2025).

circ-Amotl1 is highly expressed in neonatal human cardiac tissue, and its overexpression promotes cardiac repair in post-myocardial infarction models (Hou et al., 2019). circ-Amotl1 interacts with PDK1 and AKT1 to enhance nuclear translocation of pAKT, protecting cardiomyocytes from doxorubicin-induced cardiomyopathy (Santer et al., 2019). In exercise contexts, circ-Amotl1 contributes to cardioprotective effects through protein interactions, though specific miRNA sponging is not detailed (Hou et al., 2019). circIGF1R interacts with DDX5 to activate β-catenin signaling, promoting cardiac repair in myocardial infarction (Shan et al., 2024). circIGF1R overexpression reduces infarction size and improves cardiac function via β-catenin pathway in mice (Shan et al., 2024).

In coronary heart disease, circRNAs act as miRNA sponges to influence cardiomyocyte death, with circ_0001445 sponging miR-208a-3p to regulate autophagy (Wang et al., 2024). circ_0010729 sponges miR-187 to promote apoptosis in hypoxia (Gao et al., 2023). circ_0001274 sponges miR-138-5p to modulate inflammation (Zhang et al., 2024). These interactions in CHD models suggest potential modulation by exercise to enhance cardioprotection (Wang et al., 2024). Regular exercise alters circulating miRNAs like miR-1, miR-133a, miR-208a, with miR-133a increasing post-exercise to regulate cardiac remodeling (Denham and Prestes, 2016). Aerobic exercise training regulates cardiac miRNAs such as miR-29a and miR-30a, modulating extracellular matrix genes in HF (Peng et al., 2020). miR-222 is induced by exercise, promoting cardiomyocyte growth via PUMA inhibition (Liu et al., 2009).

**FIGURE 5:**
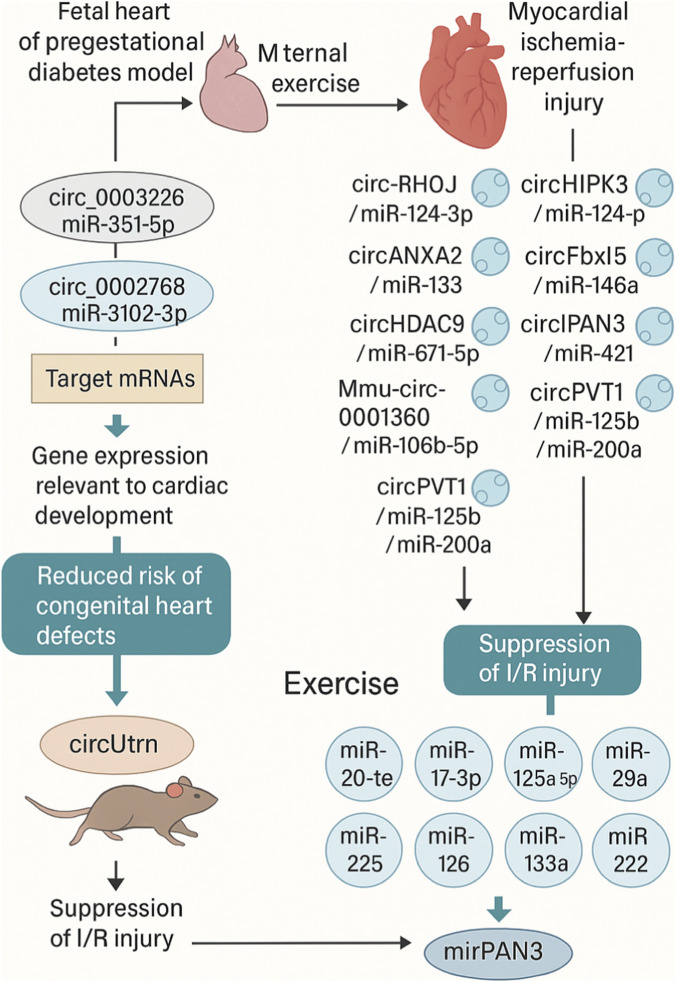
The major circRNA–miRNA regulatory networks involved in exercise-induced cardioprotection, maternal exercise–mediated fetal cardiac protection, and ischemia-reperfusion (I/R) injury modulation. In pregestational diabetes models, maternal exercise normalizes pathological circRNA–miRNA interactions in fetal hearts, including circ_0003226–miR-351-5p and circ_0015638–miR-351-5p, as well as circ_0002768–miR-3102-3p, restoring gene networks essential for cardiac morphogenesis. This normalization occurs within a regulatory system comprising 5 circRNAs, 12 miRNAs, and 28 mRNAs, which counteracts diabetes-induced dysregulation of 188 circRNAs, 57 miRNAs, and 506 mRNAs. These exercise-responsive circRNA–miRNA axes contribute to fetal cardiac protection by rescuing developmental pathways disrupted by maternal diabetes. The figure also highlights key circRNAs implicated in myocardial ischemia-reperfusion injury. Pathogenic circRNAs such as circ-RHOJ.1 (miR-124-3p/NRG-1), circANXA2 (miR-133), circHIPK3 (miR-124-3p), circ_0050908 (miR-324-5p/TRAF3), circ-0001380 (miR-106b-5p/Phlpp2), circFbxl5 (miR-146a), circHDAC9 (miR-671-5p/SOX4), circPAN3 (miR-421/Pink1), PVT1 (miR-125b, miR-200a), circFoxo3, circARAP1, and FEACR promote apoptosis, inflammation, and impaired autophagy during reperfusion injury. Conversely, cardioprotective circRNAs—including circUtrn, which is upregulated by swimming exercise and required for physiological hypertrophy—buffer miRNAs such as miR-17-3p to enhance survival and prevent I/R damage.

Exercise-induced miRNAs like miR-17-3p protect against I/R by contributing to cardiac growth (Shi et al., 2017). miR-126 is upregulated by endurance exercise, enhancing angiogenesis via VEGF signaling (Uhlemann et al., 2014). In HF, exercise restores miR-125b-3p and miR-1290, improving ejection fraction (Sanchis-Gomar et al., 2021). miR-31a-5p and miR-214-3p enhance function in hiPSC-CMs post-exercise (Plaza-Diaz et al., 2022). Exosomal miR-455-5p and miR-29b-3p reduce MMP9 in diabetes (Chaturvedi et al., 2015). miR-342-5p from heart and BAT exosomes reduces apoptosis (Zhao et al., 2022). Lung-derived miR-421-3p modulates ACE2 post-exercise exposure (Maemura et al., 2018). circRNA FEACR inhibits ferroptosis by interacting with NAMPT, alleviating I/R (Ju et al., 2023). circRNA Foxo3 acts as a regulator in cardiac I/R during transplantation (Su et al., 2021). circARAP1 promotes pro-fibrotic and apoptotic activities in I/R (Li et al., 2023). These circRNA mechanisms, often involving miRNA sponging, intersect with exercise pathways to enhance protection (Gao et al., 2023). circRNAs serve as miRNA sponges, protein scaffolds, and translation participants in CVDs (Gao et al., 2023). In CHD, circRNAs regulate cardiomyocyte death by sponging miRNAs like miR-208a-3p and miR-187 (Pan et al., 2025). Future research should validate exercise modulation of circRNA-miRNA networks in human cardiac tissues (Ye et al., 2023). Explore circUtrn’s miRNA interactions in hypertrophy models (Wang et al., 2023b). Integrate with epigenomics for personalized cardioprotection (Gao et al., 2023). Investigate miR-17-3p and circRNA sponges in exercise protocols (Shi et al., 2017). In conclusion, circRNA-miRNA interactions like circ_0003226/miR-351-5p in maternal exercise protect fetal hearts (Ye et al., 2023). circPAN3/miR-421 in I/R suppression offer targets for exercise-based therapies (Zh et al., 2021). These axes provide biomarkers for CVD management (Figure 5) (Gao et al., 2023). Due to their high stability and specific sponging capabilities, circRNAs represent a durable layer of cardioprotection, particularly in mitigating ischemia-reperfusion injury and protecting against developmental defects in diabetic pregnancies.

Exercise-induced miRNAs with known cardioprotective roles, such as miR-17-3p, miR-125a-5p, miR-20a-5p, miR-126, miR-214-3p, and miR-144, are shown linking exercise stimuli to reduced apoptosis, improved angiogenesis, enhanced autophagy, and activation of survival pathways (e.g., PTEN, NF-κB, SIRT1, TGF-β). These interactions demonstrate how exercise reshapes circRNA–miRNA–mRNA networks to attenuate myocardial injury, promote cardiac regeneration, and improve overall cardiac resilience (Table 5).

**TABLE 5:** Circular RNA–microRNA interactions in exercise-induced cardioprotection.

CircRNA	miRNA	Interaction type	Exercise context/Model	Cardioprotective effect	Mechanism/Details	References
circ_0003226, circ_0015638	miR-351-5p	Pair normalization	Maternal exercise in pregestational diabetes fetal hearts	Reverses diabetes-induced alterations; protects cardiac development	Part of 5 circRNA-12 miRNA-28 mRNA network; normalizes 188 circRNAs	Ye et al. (2023)
circ_0002768	miR-3102-3p.2-3p	Normalization	Maternal exercise in diabetic pregnancy	Reduces congenital heart defect risk; modulates morphogenesis	Targets mRNAs for cardiac gene expression; partial normalization	Ye et al. (2023)
circ-RHOJ.1	miR-124-3p	Targeting	Myocardial IRI	Regulates proliferation/apoptosis	Via NRG-1 axis; aggravates injury if dysregulated	(Xu et al., 2022)
circANXA2	miR-133	Sponging	Myocardial IRI rat model	Promotes apoptosis if upregulated	Inhibits miR-133; silencing protects	Wang et al. (2020); Zong and Wang (2020)
circHIPK3	miR-124-3p	Binding	Myocardial IRI	Aggravates injury	Sponges miR-124-3p; potential exercise modulation	Bai et al. (2019)
circ_0050908	miR-324-5p	Sponging	Myocardial IRI	Worsens damage; upregulates TRAF3	Exercise may downregulate for protection	Ghaffari-Nazari et al. (2022)
mmu-circ-0001380	miR-106b-5p	Modulation	Myocardial IRI	Attenuates injury upon knockdown	Via Phlpp2 axis; SIRT1/Nrf2 signaling	Wang et al. (2023a)
circRNA Fbxl5	miR-146a	Sponging	IRI in cardiomyocytes	Regulates apoptosis	Knockdown enhances protection	Li et al. (2022b)
circHDAC9	miR-671-5p	Regulation	IRI reperfusion	Controls proliferation/migration/apoptosis	Via SOX4 signaling axis	Liu et al. (2024)
circPAN3	miR-421	Targeting	Myocardial IRI	Suppresses autophagy; ameliorates injury	Via Pink1 axis; potential exercise target	Zhang et al. (2021)
circRNA PVT1	miR-125b, miR-200a	Targeting	Sepsis-induced kidney injury (analogous to IRI)	Prevents injury upon silencing	Regulates HMGB1; exercise may mimic	Luo et al. (2021)
circUtrn	N/A (direct)	Upregulation	Swimming-trained mouse cardiomyocytes	Required for hypertrophy; prevents IRI	Deficiency impairs adaptation; *ex vivo*/*in vivo* protection	Kühnisch et al. (2023)
circ-Amotl1	N/A (protein interaction)	Overexpression	Neonatal cardiac tissue; post-MI	Promotes repair; protects from cardiomyopathy	Interacts with PDK1/AKT1; nuclear pAKT translocation	Gao et al. (2023); Santer et al. (2019)
circIGF1R	N/A (protein)	Activation	Myocardial infarction	Reduces infarct size; improves function	Interacts with DDX5; activates β-catenin	Shan et al. (2024)
circ_0001445	miR-208a-3p	Sponging	Coronary heart disease	Regulates autophagy; influences cell death	Potential exercise modulation in CHD	Wang et al. (2024)
circ_0010729	miR-187	Sponging	Hypoxia in CHD	Promotes apoptosis	Exercise may downregulate	Gao et al. (2023)
circ_0001274	miR-138-5p	Sponging	Inflammation in CHD	Modulates inflammatory responses	Potential for exercise-based protection	Zhang et al. (2024)

## 8 Exosomal microRNAs: central mediators of exercise-induced cardioprotection

Exosomal microRNAs (miRNAs) are small non-coding RNAs packaged within exosomes, EVs of 30–200 nm in size that facilitate intercellular communication by transferring molecular cargo between cells and tissues. In the realm of exercise physiology, these exosomal miRNAs emerge as pivotal mediators of cardioprotection, orchestrating systemic adaptations that shield the heart from stressors like ischemia-reperfusion injury (IRI), myocardial infarction (MI), and chronic cardiovascular diseases (CVDs). Released in response to physical activity—particularly aerobic exercises such as running or swimming—exosomes originate from diverse sources including skeletal muscle, adipose tissue, endothelial progenitor cells (EPCs), and cardiomyocytes. Their miRNA cargo, selectively enriched during exercise, modulates target gene expression in recipient cardiac cells, suppressing apoptosis, inflammation, and fibrosis while promoting angiogenesis, mitochondrial biogenesis, and metabolic shifts. For example, exercise elevates exosomal miR-342-5p from plasma, which targets pro-apoptotic genes like Caspase-9 and JNK2, enhancing cell survival via Akt signaling (Hou et al., 2019). This paracrine mechanism exemplifies how exosomal miRNAs amplify exercise’s remote effects, bridging organs like muscle and heart to foster resilience against CVD, with potential as biomarkers for fitness monitoring or therapeutic mimics in sedentary populations.

In rodent models of MI, long-term exercise training induces the release of exosomal miR-342-5p, which confers protection by attenuating apoptosis and oxidative stress in cardiomyocytes. Experimental evidence shows that exosomes from exercise-trained rats, when infused into MI models, reduce infarct size and improve ejection fraction through miR-342-5p-mediated suppression of Ppm1f, thereby activating Akt pathways (Hou et al., 2019). Similarly, brown adipose tissue (BAT)-derived exosomes enriched with miR-125b-5p, miR-128-3p, and miR-30d-5p target MAPK signaling components like Map3k5 and Map2k7, inhibiting caspase-3 activation and IRI (Zhao et al., 2022). These miRNAs are upregulated post-exercise, highlighting adipose tissue’s role in systemic cardioprotection. Adipose-derived exosomal miR-17-3p, induced by exercise, targets calcium/calmodulin-dependent protein kinase II (CaMKII), suppressing the RIPK3/MLKL pathway to alleviate programmed necrosis in IRI models. BAT ablation abolishes this effect, confirming tissue-specific origins (Lai et al., 2023). In diabetic cardiomyopathy, exercise-derived exosomes carry miR-455-5p and miR-29b-3p, downregulating MMP9 to mitigate fibrosis and improve myocyte coupling (Chaturvedi et al., 2015). EPC-derived exosomal miR-126 activates PI3K/Akt to upregulate BDNF and VEGF, fostering angiogenesis in ischemic hearts (Han et al., 2020). Skeletal muscle releases exosomes with miR-130a, which modulates ROS/NF-κB in endothelial cells, countering capillary rarefaction in obesity (Fernandes et al., 2018). Liver-derived exosomal miR-122-5p targets AGPAT1 to enhance fatty acid utilization and VEGF expression, protecting against endothelial dysfunction (Lai et al., 2023). Cerebrospinal fluid exosomes post-exercise carry miR-138-5p, suppressing LCN2 in astrocytes to reduce inflammation in stroke models (Lai et al., 2023). Cardiomyocyte-derived cardiosomes enriched with miR-455 post-exercise inhibit MMP9, reducing fibrosis in diabetic hearts (Chaturvedi et al., 2015). miR-10b-5p in exercise EVs targets Mib1 to activate Notch signaling, promoting angiogenesis (Lai et al., 2023). miR-29b and miR-455–1 suppress fibrotic genes in cardiac fibroblasts (Chaturvedi et al., 2015).

**FIGURE 6:**
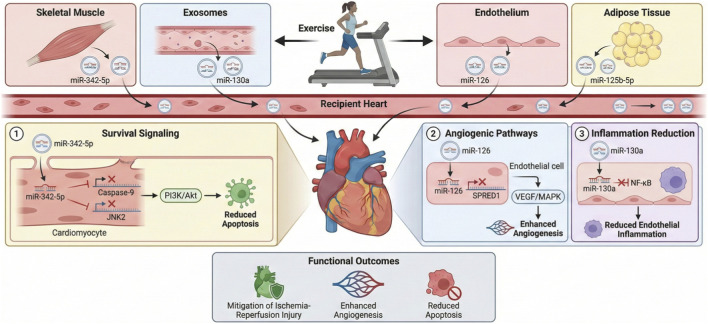
The central role of exercise-induced exosomal microRNAs (miRNAs) in mediating systemic and cardiac protection across diverse physiological and pathological contexts. During physical activity, particularly endurance and aerobic exercise, exosomes are released from skeletal muscle, brown adipose tissue (BAT), endothelial progenitor cells (EPCs), liver, cardiomyocytes, and the central nervous system. These 30–200 nm vesicles encapsulate selectively enriched miRNAs that travel through circulation to the heart, where they modulate apoptosis, inflammation, fibrosis, angiogenesis, mitochondrial function, and metabolic remodeling.

Mechanistically, exercise stimulates exosome biogenesis via increased TSG101, CD63, and CD81 expression, with cargo like miR-342-5p sponging targets to derepress survival pathways (Hou et al., 2019). In IRI, exosomal miR-17-3p inhibits CaMKII/RIPK3/MLKL, reducing necrosis (Lai et al., 2023). Meta-analyses show exercise exosomes reduce apoptosis (SMD -3.80), infarct volume (5.16), and boost angiogenesis (2.63) and ejection fraction (8.13) (Lai et al., 2023). miR-126-3p targets SPRED1/PIK3R2 for angiogenesis in diabetes (Han et al., 2020). miR-21a-5p regulates lipid metabolism via FABP7/HMGCR (Lee et al., 2020). miR-133a-5p targets CASP3/8/9 to reduce apoptosis (Peng et al., 2020). miR-29a-3p inhibits TGF-β1/SMAD2/3 for antifibrosis (Wang et al., 2018). miR-222-3p promotes growth via HIPK1/P27 (Liu et al., 2009). miR-17-3p targets TIMP-3/PTEN for hypertrophy (Shi et al., 2017). miR-486-5p inhibits PTEN/FOXO1 for IRI protection (Li et al., 2018). miR-342-5p targets CASP9/JNK2 for IRI attenuation (Hou et al., 2019). In HF, exercise restores exosomal miR-125b-3p/miR-1290, improving ejection fraction (Sanchis-Gomar et al., 2021). miR-31a-5p/miR-214-3p enhance hiPSC-CM function (Plaza-Diaz et al., 2022). In obesity, miR-16-5p/miR-208a-3p downregulation restores VEGF (Fernandes et al., 2018). Interorgan crosstalk involves heart/BAT exosomal miR-342-5p reducing apoptosis (Hou et al., 2019). Lung-derived miR-421-3p modulates ACE2 (Maemura et al., 2018). Therapeutic trials target miR-132-3p/miR-92a-3p (Gallant‐Behm et al., 2018; Täubel et al., 2021). Implications for therapy include exosomal miRNA mimics for CVD, with exercise protocols optimizing cargo (Lai et al., 2023). In MI, exosomal miR-25-3p from MSCs alleviates damage by targeting EZH2 (Peng et al., 2020). Future directions: Validate in humans, explore miR-3084b-3p (Wang et al., 2024). Integrate omics for personalized exercise (Correia et al., 2021). In conclusion, exosomal miRNAs like miR-342-5p and miR-17-3p are central to exercise-induced cardioprotection, regulating apoptosis and angiogenesis (Hou et al., 2019; Lai et al., 2023). These offer biomarkers and targets for CVD (Figure 6; Table 5) (Zhang et al., 2024). It is crucial to note that many cited studies isolate exosomes using precipitation-based kits, which often co-isolate non-vesicular proteins and lipoproteins. Consequently, the attribution of functional effects solely to ‘exosomal' miRNAs in these models may be confounded by soluble factors. Future studies utilizing size-exclusion chromatography or immuno-affinity capture are required to definitively assign cardioprotective functions to specific vesicle subpopulations.

Exercise-derived exosomal miR-342-5p from plasma suppresses pro-apoptotic targets such as Caspase-9 and JNK2, enhancing Akt-mediated survival signaling and reducing ischemia-reperfusion injury (IRI). BAT-derived exosomes enriched with miR-125b-5p, miR-128-3p, and miR-30d-5p inhibit MAPK pathway components to decrease caspase-3 activation and oxidative stress. Exosomal miR-17-3p, also induced by exercise, downregulates CaMKII and the RIPK3/MLKL necroptosis pathway, protecting the myocardium against reperfusion injury. EPC-derived miR-126 promotes angiogenesis by activating PI3K/Akt and upregulating VEGF and BDNF in ischemic cardiac tissue. Skeletal muscle contributes miR-130a-containing exosomes that attenuate ROS-induced endothelial dysfunction via inhibition of NF-κB signaling. Liver-derived exosomal miR-122-5p enhances fatty acid utilization and endothelial stability through targeting AGPAT1. Exercised cardiomyocytes release miR-455 and miR-29b-3p, which suppress MMP9 expression and reduce fibrosis, particularly in diabetic cardiomyopathy (Table 6). A major limitation in current research is the difficulty in definitively identifying the tissue origin of circulating exosomes. Plasma contains a mixed pool of vesicles from platelets, erythrocytes, leukocytes, and endothelium, which often masks the smaller contribution from skeletal muscle or the heart. While some studies use tissue-specific surface markers (e.g., SGCA for muscle, CD31 for endothelium) to immunocapture specific vesicle subpopulations, these methods are not yet standardized. Consequently, many ‘exercise-induced' miRNAs described in literature may originate from the immune system’s response to exercise rather than from the contractile tissue itself, confounding the understanding of muscle-heart crosstalk.

**TABLE 6:** Exosomal microRNAs: central mediators of exercise-induced cardioprotection.

Exosomal miRNA	Source tissue	Exercise type/Model	Target genes/Pathways	Cardioprotective effect	Mechanism/Details	References
miR-342-5p	Plasma/Heart/BAT	Long-term training in MI/IRI rats	Caspase-9, JNK2, Ppm1f	Attenuates apoptosis/oxidative stress; reduces infarct size	Activates Akt; exosome infusion improves ejection fraction	Hou et al. (2019)
miR-125b-5p, miR-128-3p, miR-30d-5p	Brown Adipose Tissue (BAT)	Exercise in IRI models	Map3k5, Map2k7 (MAPK); Caspase-3	Inhibits necrosis; alleviates IRI	BAT ablation abolishes effect; suppresses RIPK3/MLKL	Zhao et al. (2022)
miR-17-3p	Adipose	Exercise-induced in IRI	CaMKII, RIPK3/MLKL	Suppresses programmed necrosis	Targets calcium signaling; enhances survival	Lai et al. (2023)
miR-455-5p, miR-29b-3p	Cardiomyocytes/Cardiosomes	Exercise in diabetic cardiomyopathy	MMP9	Mitigates fibrosis; improves myocyte coupling	Downregulates fibrotic genes; antifibrotic in fibroblasts	Chaturvedi et al. (2015)
miR-126	Endothelial Progenitor Cells (EPCs)	Aerobic exercise in ischemic hearts	PI3K/Akt, BDNF, VEGF	Fosters angiogenesis; protects endothelium	Upregulates growth factors; improves vascular function	Han et al. (2020)
miR-130a	Skeletal Muscle	Exercise in obesity models	ROS/NF-κB	Counters capillary rarefaction; reduces inflammation	Modulates endothelial ROS; systemic vascular protection	Fernandes et al. (2018)
miR-122-5p	Liver	Post-exercise	AGPAT1, VEGF	Enhances fatty acid utilization; protects endothelium	Improves metabolic shift; angiogenesis promotion	Lai et al. (2023)
miR-138-5p	Cerebrospinal Fluid	Exercise in stroke models	LCN2	Reduces inflammation in astrocytes	Suppresses neuroinflammation; indirect cardiac benefits	Han et al. (2020)
miR-10b-5p	Exercise EVs	General exercise	Mib1, Notch signaling	Promotes angiogenesis	Activates Notch; vascular remodeling	Lai et al. (2023)
miR-126-3p	Plasma/Exosomes	Endurance in diabetes	SPRED1/PIK3R2	Enhances angiogenesis	VEGF signaling activation; reduces IRI	Han et al. (2020)
miR-21a-5p	Exosomes	Training in lipid models	FABP7/HMGCR	Regulates lipid metabolism	Reduces cholesterol accumulation; metabolic protection	Wang et al. (2020)
miR-133a-5p	Muscle-derived	Aerobic training	CASP3/8/9	Reduces apoptosis	Caspase inhibition; improves remodeling	Peng et al. (2020)
miR-29a-3p	Exosomes	Exercise in MI	TGF-β1/SMAD2/3	Inhibits fibrosis	Suppresses TGF-β signaling; antifibrotic	Wang et al. (2018)
miR-222-3p	Plasma	Post-exercise	HIPK1/P27	Promotes cardiomyocyte growth	Cell cycle regulation; hypertrophy protection	Liu et al. (2009)
miR-17-3p	Exosomes	Exercise-induced	TIMP-3/PTEN	Induces hypertrophy; protects IRI	PTEN inhibition; growth signaling	Shi et al. (2017)
miR-486-5p	Cardiomyocytes	Upregulated in IRI	PTEN/FOXO1	Protects against IRI	FOXO1 suppression; anti-apoptotic	Li et al. (2018)
miR-342-5p	BAT/Heart	Exosomal in IRI	CASP9/JNK2	Attenuates IRI	JNK inhibition; Akt activation	Hou et al. (2019)
miR-125b-3p, miR-1290	Circulating Exosomes	Exercise in HF	Various (ejection fraction-related)	Improves ejection fraction	Restores cardiac function; remodeling	Sanchis-Gomar et al. (2021)
miR-31a-5p, miR-214-3p	hiPSC-CMs	Post-exercise	Maturation genes	Enhances CM function	Improves contractility; maturation	Plaza-Diaz et al. (2022)
miR-16-5p, miR-208a-3p	Exosomes in obesity	Exercise restoration	VEGF-related	Restores angiogenesis; prevents hypertrophy	Downregulation corrects dysfunction	Fernandes et al. (2018)
miR-421-3p	Lung-derived	Post-exposure exercise	ACE2	Modulates ACE2 expression	Renin-angiotensin protection	Maemura et al. (2018)
miR-25-3p	MSC Exosomes	Mimicking exercise in MI	EZH2	Alleviates MI damage	Epigenetic regulation; repair	Peng et al. (2020)

## 9 Integrated mechanistic framework and future directions

### 9.1 A unified hierarchical model of cardioprotection

Based on the synthesized data, we propose a hierarchical model of exercise-induced cardioprotection. In this framework, Acute Exercise acts as a ‘molecular shock,' triggering the rapid release of exosomal miRNAs (e.g., miR-342-5p, miR-133a) from skeletal muscle and endothelium to serve as immediate systemic signals. Upon reaching the myocardium, these exerkines initiate a secondary wave of regulation where lncRNAs (e.g., MALAT1, H19) function as ‘chromatin scaffolds' or ‘sponges' to fine-tune the stability and availability of these miRNAs. Finally, Chronic Training consolidates these transient signals into stable phenotypic adaptations (angiogenesis, mitochondrial biogenesis) through the durable downregulation of pathological drivers like miR-208a and the upregulation of protective circRNAs (circUtrn). This multi-layered architecture ensures that the heart’s adaptive response is both rapid (miRNA-mediated) and sustained (lncRNA/circRNA-mediated).

### 9.2 Current research gaps

Despite remarkable advances in understanding how exercise shapes non-coding RNA (ncRNA) biology, substantial gaps still hinder the translation of these discoveries into clinically meaningful strategies for cardiovascular protection. Although numerous studies have documented striking changes in miRNAs, lncRNAs, circRNAs, and exosomal ncRNAs following exercise, much of the existing evidence remains largely associative. Many ncRNAs correlate with improved cardiac function, reduced fibrosis, enhanced angiogenesis, or greater metabolic resilience after exercise, but few have been rigorously tested to determine whether they are truly necessary or sufficient to mediate these beneficial adaptations. Most mechanistic experiments rely on *in vitro* systems or non-exercise injury models and do not adequately reflect the dynamic, systemic, and multi-organ environment triggered by real exercise. Gain- or loss-of-function studies integrated directly into exercise protocols are critically needed to differentiate ncRNAs that simply respond to exercise from those that act as genuine molecular drivers of cardioprotection.

Another major gap lies in the limited mapping of integrated ncRNA networks. Exercise affects thousands of transcripts, yet research often focuses on isolated axes—such as MALAT1/miR-150-5p, H19/miR-139, GAS5/miR-217, or circUtrn-related circuits—without situating them within the broader regulatory ecosystem that includes transcription factors, RNA-binding proteins, epigenetic modifiers, and downstream protein targets. Because ncRNAs frequently act in overlapping, redundant, or competing pathways, studying them in isolation oversimplifies the biology. There is an urgent need for multi-omics approaches that profile transcriptomic, epigenomic, proteomic, and metabolic responses within the same subjects before and after exercise. Computational modeling and systems biology will be required to reconstruct high-resolution ncRNA networks and identify core nodes that truly orchestrate adaptive remodeling. Perturbation-based experiments, where multiple ncRNAs are modulated simultaneously, will further illuminate network robustness and vulnerability.

A closely related challenge is the incomplete understanding of how exercise modality, intensity, timing, and duration shape ncRNA regulation. Current evidence suggests that endurance training, resistance exercise, high-intensity interval training, and acute vs. chronic stimuli activate distinct ncRNA signatures. However, most studies use narrow, single-modality protocols that prevent establishment of dose–response relationships. Without systematic comparisons of frequency, intensity, time, and type of exercise, it is impossible to determine optimal “exercise prescriptions” for triggering specific ncRNA-mediated benefits. Time-course studies are also lacking; few investigations evaluate the immediate early responses within minutes or hours of exercise, the intermediate adaptations over days or weeks, or the long-term remodeling that unfolds across months. The reversibility of exercise-induced ncRNA changes after detraining, and the possibility that some ncRNAs encode a form of “molecular memory” that influences future cardiac resilience, remain almost entirely unexplored.

Human relevance represents another underdeveloped area. Much of the mechanistic understanding comes from rodent models, which do not fully recapitulate human physiology, comorbidities, or training patterns. Data on exercise-responsive lncRNAs and circRNAs in humans are particularly sparse. To translate these findings, large-scale, longitudinal human studies are necessary, ideally involving standardized exercise interventions combined with cardiac imaging, VO_2_max testing, blood sampling, and serial profiling of circulating and exosomal ncRNAs. These studies must include diverse populations across age, sex, ethnicity, baseline fitness, and disease states to ensure broad applicability. It will be essential to correlate ncRNA signatures not only with intermediate markers like ventricular remodeling or endothelial function but also with hard outcomes such as HF hospitalization, arrhythmias, myocardial infarction, or cardiovascular mortality.

Technical challenges pose another substantial barrier. Exosomal miRNAs are central to the concept of ncRNA-mediated inter-organ communication, yet methodological inconsistencies persist across studies. There is no universally accepted standard for isolating EVs, normalizing miRNA measurements, or quantifying lncRNAs and circRNAs in circulation. Differences in EV isolation methods, RNA extraction, sequencing platforms, and normalization strategies can produce dramatically different results. To enable reliable biomarker discovery, the field must adopt harmonized protocols for exosome characterization, RNA quantification, and reporting standards. Emerging technologies such as single-vesicle analysis, spatial transcriptomics, and cell-specific RNA tracking will be crucial for identifying the exact tissues that release or take up specific ncRNAs during and after exercise.

Individual-level modifiers—including sex, age, hormonal status, and training background—introduce additional complexity. Evidence suggests that males and females exhibit different ncRNA responses to both acute and chronic exercise, likely due to sex hormones, sex-specific epigenetic landscapes, and differences in muscle mass and metabolic signaling. Aging alters miRNA biogenesis machinery, mitochondrial function, and extracellular vesicle production, potentially changing the magnitude and direction of exercise-induced ncRNA shifts. Training status shapes baseline ncRNA expression, meaning that sedentary individuals, recreational athletes, and elite endurance or strength athletes may exhibit substantially different profiles. Most studies fail to incorporate these factors as biological variables, highlighting the need for better stratified analyses and adequately powered subgroup studies. Understanding how these modifiers shape ncRNA-mediated cardioprotection will be essential for future personalized exercise recommendations.

Therapeutic targeting of ncRNAs offers enormous promise but remains in its infancy. Although miRNA mimics and inhibitors have shown beneficial effects in preclinical models, challenges related to off-target activity, delivery efficiency, tissue specificity, and long-term safety remain unresolved. LncRNAs and circRNAs pose even greater difficulty because of their structural complexity, diverse mechanisms, and widespread genomic interactions. Targeted delivery systems—such as engineered exosomes, cardiac-specific nanoparticles, viral vectors with cardiomyocyte-selective promoters, and peptide-based homing technologies—must be further refined to minimize systemic exposure and avoid unintended immune or oncogenic consequences. Importantly, ncRNA therapeutics may not need to fully mimic exercise; rather, they may serve as adjuncts that enhance or extend exercise-induced cardioprotection, especially in individuals unable to tolerate high-intensity training.

### 9.3 Translational challenges: from cage to bedside

Translating these ncRNA mechanisms to human heart failure (HF) patients faces significant hurdles regarding exercise dose and intensity. Rodent studies typically employ forced, high-intensity treadmill running that mimics elite athletic training—a regimen often unfeasible for HF patients with reduced ejection fraction and comorbidities.

- Intensity Thresholds: It remains unknown whether low-to-moderate intensity exercise (e.g., walking), which is feasible for most HF patients, is sufficient to trigger the release of critical exerkines like exosomal miR-126 or miR-133a.
- Feasibility: In patients with advanced HF (NYHA Class III-IV), the skeletal muscle myopathy (‘cardiac cachexia') may severely blunt the muscle’s ability to secrete protective exosomes, potentially rendering exercise less effective. This ‘exerkine resistance' suggests that for some patients, ncRNA-based therapeutics (mimics) may be necessary to bypass the need for vigorous physical activity.

### 9.4 Future directions: a roadmap to precision exercise medicine

To transition ncRNA biology from molecular observation to clinical utility, research must pivot toward three high-priority pillars.

1. Establishing Causal Signatures: Move beyond correlational “profiling” to prioritize gain- and loss-of-function studies within exercise protocols. The clinical focus should be on validating the miR-132 axis (currently in Phase 1b trials) as a primary target for pharmacological exercise mimetics.
2. Mapping Multi-Organ Crosstalk: Deconvolute the “exerkine” signal by utilizing single-vesicle analysis and spatial transcriptomics. Identifying the precise origin and cardiac uptake of muscle-derived exosomes (e.g., via the PGC-1$\alpha$ pathway) is essential for designing heart-specific delivery systems.
3. Technological standardization: Rapidly adopt harmonized protocols for extracellular vesicle (EV) isolation and ncRNA normalization. Establishing robust, sex-stratified reference intervals is a prerequisite for using circulating ncRNAs as real-time biomarkers for training efficacy and maladaptation

Integrating these molecular insights with machine-learning models—combining ncRNA data with wearable sensor outputs and clinical imaging—will eventually enable dynamic, adaptive exercise programming tailored to an individual’s unique molecular landscape.

## 10 Conclusion

The evidence of the past decade establishes ncRNAs—specifically miRNAs, lncRNAs, and circRNAs—as the central executors of exercise-induced cardioprotection. Rather than passive biomarkers, these molecules are active mediators that orchestrate angiogenesis, mitochondrial rejuvenation, and anti-fibrotic remodeling.

Significant milestones have been reached in identifying key “myomiR” signatures (e.g., miR-1, miR-133a) and the transformative role of exosomal communication in systemic health. However, the field now faces a “translational bottleneck” characterized by methodological inconsistencies, a lack of causal validation in human cohorts, and undifferentiated approaches to heart failure phenotypes (HFrEF vs. HFpEF).

Realizing the full potential of ncRNA biology requires a shift toward systems-level investigations that account for biological variables such as sex, age, and disease etiology. By closing the gap between basic mechanistic discovery and standardized clinical application, ncRNA-based diagnostics and therapeutics can democratize the protective benefits of exercise—extending cardiac resilience even to those unable to perform physical activity.
